# Proton Therapy in The Treatment of Head And Neck Cancers- Review

**DOI:** 10.1007/s11912-024-01592-9

**Published:** 2024-08-08

**Authors:** Kamila Bała, Yana Samovich, Karolina Dorobisz

**Affiliations:** Department of Otolaryngology, Head and Neck Surgery, Wroclaw Medical University, Borowska 213, 50-556, Wrocław, Poland

**Keywords:** Proton Therapy, Proton Beam Therapy, Head and Neck Cancer, Cancer Proton Therapy, Toxicity

## Abstract

**Purpose of Review:**

Head and neck cancers rank as the seventh most common cancer worldwide, nearly half of which result in death. The most common treatment methods for head and neck cancers include radiotherapy and surgery. Proton therapy has emerged in radiotherapy for cases where tumors are located near anatomically sensitive areas where the radiation dose must be strictly limited. The purpose of the work is to discuss the role of the proton therapy in the treatment in various types of cancer, and particularly head and neck tumors.

**Recent Findings:**

Proton therapy allows for the delivery of radiation doses to critical organs to be reduced, resulting in a decrease in the occurrence of late adverse effects on these organs. The occurrence of side effects caused by proton therapy depends on the relative and absolute volume of organs at risk receiving specific radiation doses.

**Summary:**

Proton therapy represents a promising alternative to conventional radiotherapy due to the reduced number of complications in healthy tissues by delivering a lower radiation dose outside the tumor area.

## Introduction

According to GLOBOCAN 2020, head and neck cancers rank as the seventh most common cancer worldwide, accounting for over 900,000 new cases annually, nearly half of which result in death. Currently, the treatment strategies for head and neck cancers encompass various disciplines including surgery, radiotherapy, chemotherapy, and supportive care [1]. The most common treatment methods for head and neck cancers include radiotherapy, surgery, or a combination of both [2]. Due to distant metastases being less common in head and neck cancers compared to other malignancies, the primary determinant of patient survival is the local growth of the tumor, necessitating effective locoregional control [2].

The concept of applying proton beams in clinical practice was pioneered by the American physicist Robert Wilson in 1946 [3]. The first clinical application, involving the removal of pituitary tumors in breast cancer patients, took place in Berkeley, California in 1954. This invention led to proton therapy being administered to nearly 40,000 patients over half a century [2]. The unique physical properties of proton therapy allow for the delivery of higher radiation doses while simultaneously reducing the radiation dose to surrounding tissues, resulting in fewer complications [4].

## Study Design

The purpose of the work is to discuss the role of the proton therapy in the treatment in various types of cancer, and particularly head and neck tumors. The literature search strategy was carried out using the PubMed base based on the keyword combination: Proton Therapy, Proton Beam Therapy, Head and Neck Cancer, Cancer Proton Therapy, Toxicity. The references of the publications of interest were also screened for relevant papers. There were no limitations in regard to the publication date.

## Proton Therapy

**Fig. 1:**
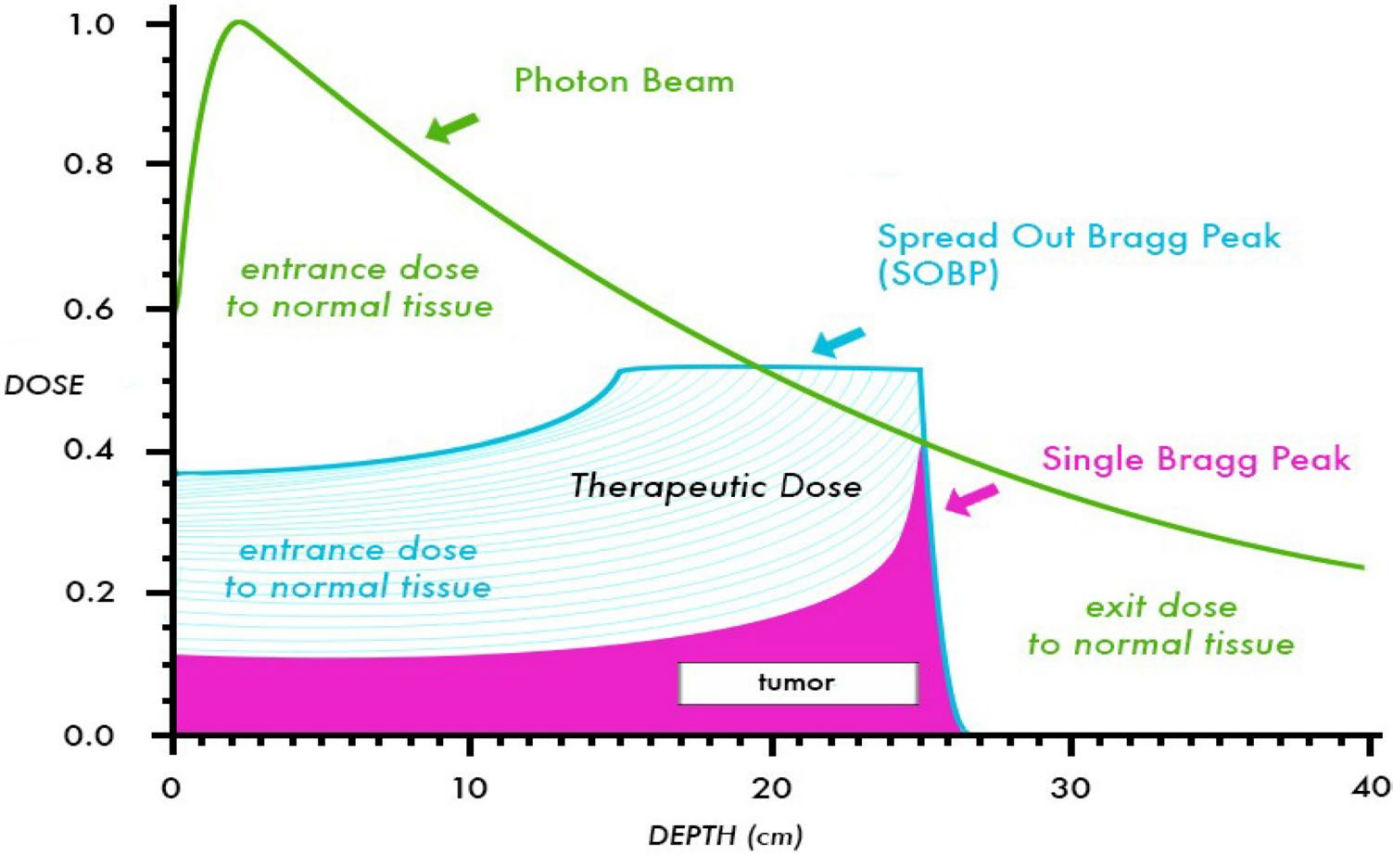
Comparison of depth dose curves for a 10 MV photon beam and a 10 MeV proton beam (shown with and without a SOBP). This figure shows the decreased entrance dose and absence of exit dose for the proton beam in comparison to the photon beam. [15] –with the author's permission Torunn I. Yock

Proton therapy has emerged in radiotherapy for cases where tumors are located near anatomically sensitive areas [5]. Examples include tumors at the base of the skull, spine or its vicinity close to the brainstem, cranial nerves, and/or optic structures, as well as other tumors such as hepatocellular carcinoma to maximize the preservation of non-cancerous liver parenchyma or mediastinal lymphomas, which occupy a large area, and many more [6]. Maximizing the therapeutic ratio is the primary goal of radiotherapy, involving delivering lethal doses of ionizing radiation to the tumor while minimizing the dose to normal tissues [6]. In proton therapy, the beam energy can be directed to a precise depth, resulting in reduced toxicity compared to conventional photon radiation, as outlined in Table 1. This is achievable because proton beams deposit most of their energy at the end of their path as their velocity decreases, ultimately releasing all their energy, thus lacking an exit dose [7]. Consequently, the radiation dose increases to a certain peak, then sharply decreases and is not delivered beyond a certain range [7]. The position of the maximum dose deposition is termed the Bragg peak, illustrated in Fig. 1. In clinical proton therapy, a spread-out Bragg peak (SOBP) is commonly used to achieve tumor volume coverage [8]. The SOBP is a sum of several Bragg peaks with different ranges. In contrast, X-rays utilize photons, which enter and pass through tissues, depositing energy and exiting the body on the other side, constituting an exit dose that affects nearby tissues [7].

**Table 1:** Comparison of Proton Therapy with Conventional Photon Radiation

Feature	Proton Therapy	Conventional Photon Radiation
Type of radiation	Ionizing radiation	Ionizing radiation
Cellular products	Free radicals and reactive oxygen species	Free radicals and reactive oxygen species
Particles utilized	Protons (large particle, positive charge)	Photons (massless, uncharged)
Particle acceleration	Cyclotron	Linear accelerator
Range of initial dose	No initial dose	360 degrees
Adjacent tissues	Receive significantly lower total dose	Receive a significant total dose—larger volume of radiation with smaller dose

Based on: [6, 7]

In proton therapy, a significant group of patients comprises young individuals, especially children, adolescents, and young adults, due to the lower likelihood of complications to normal tissues with this therapy [6]. In the UK, a foreign proton therapy program was launched in 2008, with 1144 patients approved for therapy [9]. Pediatric patients represent 65% of all enrolled patients, while adolescents and young adults account for 12%. Proton therapy is likely to reduce the risk of growth and development disorders, endocrinological dysfunction, reduced fertility, and secondary tumors in children, adolescents, and young adults [10]. Another significant group comprises pregnant patients with brain tumors or tumors within the head and neck region. Study results show that the average measured equivalent dose to the fetus, considering photons and neutrons, for brain plans was 0.4 mSv for pencil beam scanning proton therapy (PBS-PRT) and 7 mSv for conventional radiotherapy (XRT) [11]. For head and neck plans, the values were 6 mSv and 90 mSv for PBS-PRT and XRT, respectively [11]. This represents a 15-fold reduction in fetal dose for head and neck tumors and a 17.5-fold reduction for brain tumor locations.

To maximize the benefits of proton therapy in preventing late radiation side effects compared to photon therapy, appropriate patient selection is necessary [12]. Various tools are used for this purpose, including Normal Tissue Complication Probability (NTCP) models, cost-effectiveness modeling, or radiation dose comparisons [13]. The most commonly reported clinical indication for patient selection for proton therapy was head and neck cancer (48% of studies), where dose comparison methods were most frequently employed [13]. In patient groups with head and neck tumors, the changing anatomy of the patient during proton therapy is of great importance. Patient anatomical changes concerning changes in tumor volume and the volume of surrounding normal tissues translate into a significant difference between the planned and actually delivered dose range. Protons are highly sensitive to tissue inhomogeneities along the beam path, and therefore, patient configuration errors, in some cases, can seriously jeopardize the proper delivery of the intended radiation dose, thus complicating locoregional control [14].

### Application of Proton Therapy in *Cancer* Treatment

Since the advent of proton therapy, the number of patients receiving such treatment has been gradually increasing worldwide [16]. The intensive advancement of science provides increasing evidence of the effectiveness of proton therapy, leading to continuous expansion of indications for the use of this therapy. Regarding pediatric patients, proton therapy is indicated for the majority of benign and malignant tumors in children: chordomas and chondrosarcomas within the skull base or spine, nasopharyngeal carcinomas, meningiomas, Ewing's sarcomas, intracranial embryonal tumors, rhabdomyosarcomas, optic nerve tumors, and other low-grade gliomas [13]. Due to the physical properties of protons and well-documented results of proton therapy, there is consistency among various foreign centers regarding this matter [17]. However, in the case of adult patients, their selection for proton therapy is variable. Indications for proton therapy in the UK, United States, Canada, the Netherlands, Australia, and New Zealand were consistent only for chordomas and chondrosarcomas within the skull base or spine [13]. This difference arises from insufficient scientific evidence confirming greater benefits of proton therapy compared to conventional photon radiation in the adult patient group.

Nevertheless, the utilization of proton therapy for extracranial tumors is increasing worldwide with the emergence of new studies in this area. In prostate cancer, proton therapy and intensity-modulated photon therapy have shown very good and comparable long-term disease control outcomes [18]. Data on reduced rates of secondary malignant tumors in the proton therapy-treated group have also been obtained, although they are purely hypothetical and require further investigation [18]. Results comparing both methods in the treatment of uterine cancer indicate that proton therapy may reduce the frequency of diarrhea at the end of radiotherapy and lower the risk of fecal incontinence after 12 months compared to intensity-modulated photon therapy [19]. In a meta-analysis evaluating the effectiveness and safety of proton and photon therapy in esophageal cancer patients, proton therapy was associated with reduced doses to at-risk organs, lower toxicity, and improved prognosis compared to photon therapy [4]. These presented results are very promising but should be further confirmed in future randomized clinical trials. Selected research outcomes of proton therapy application in liver, esophageal, lung, bladder, kidney, biliary tract, and rectal cancers are presented in Table 2 [20–29].

**Table 2:** Selected Outcomes After Proton Therapy

First author, year of publication	Disease and its location	Stage of disease—range	Number of patients	Dose range	Overall Survival (OS) rate	Progression-Free Survival (PFS) rate	Local Control (LC) rates	Toxicity grade ≥ 3
Yamaguchi, 2023^[26]^	Recurrence of liver metastases in esophageal cancer	IB—IVB	7	63.8–80 Gy /8–38 fr	1-, 2- and 3-year were 100%, 57.1% and 42.9%	1-, 2- and 3- year were all 28.6%	1-, 2- and 3- year were all 100%	Grade 3 liver abscess: 14.3%
Ono, 2020^[20]^	Esophageal squamous cell carcinoma	I – IV	54	72.0 – 90.8 Gy (RBE)	2-, 3- and 5-year were 74.9% 66.2% and 56.2%	NA	3- and 5- year were 71.5% and 61.8%	Grade 3 esophageal ulcers: 5.6%
Hiroshima, 2023^[21]^	Recurrence of lymph node metastases in esophageal cancer	NA	11	50–66 Gy / 25–33 fr	2-year was 48,0%	2- year was 27,3%	2- year was 84,6%	Grade 3 leukopenia: 27.3%
Nakamura, 2022^[22]^	Non-small cell lung cancer	Tis—T3	68 (< 80 years old) 42 (≥ 80 years old)	66–80 GyE/ 10–25 fr	3-year was 79,8% (≥ 80 years old) and 80,5% (< 80 years old)	3- year was 73,9% (≥ 80 years old) and 61,2% (< 80 years old)	NA	Grade 3 radiation pneumonitis: 4.4% (< 80 years)
Mizumoto, 2023^[23]^	Hepatocellular carcinoma	I—IV	576	66–76 Gy (RBE) / 10–22 fr	1-, 2-, 3- and 4-year were 83.8%, 68.5%, 58.2% and 50.1%	1-, 2-, 3- and 4- year were 55.2%, 37.5%, 30.2% and 22.8%	NA	Liver failure; grade 3, 4 and 5 skin inflammation, and other grade 3, e.g., gastrointestinal disorders: 4.7%
Iizumi, 2023^[24]^	Hepatocellular carcinoma with bile duct invasion	NA	15	72.6 Gy (RBE) / 22 fr	1-, 2- and 3-year were 80.0%, 58.7% and 40.2%	1-, 2- and 3- year were 72.7%, 9.7% and 0.0%	1-, 2- and 3- year were 93.3%, 93.3% and 74.7%	Acute, grade 3 bile duct inflammation: 6.7% Late, grade 3 gastric bleeding, pleural effusion: 13.3%
Araya, 2023^[25]^	Infiltrating bladder cancer	T2a-T4a	36	59.8–77.7 Gy (RBE) / 30–37 fr	3-year was 90.8%	3- year was 71.4%	3- year was 84.6%	Grade 3 urinary tract obstruction: 2.8%
Ishikawa, 2023^[27]^	Renal cell carcinoma	T1a-T3	16	66–77 Gy (RBE) / 10–35 fr	3- year was 100%	3- year was 88.9%	3- year was 94.4%	No grade 3 gastrointestinal toxicity observed
Yamazaki, 2022^[28]^	Intrahepatic and extrahepatic bile duct cancer	NA	150	44–77 Gy / 10–38 fr	1- and 2-year were 72.8% and 44.8%	1- and 2- year were 54.1% and 35.8%	1- and 2- year were 89.7% and 78.2%	Acute, grade 3 bile duct narrowing: 2.2% Late, grade 3 and above: perforation, duodenal bleeding, liver failure, bile duct narrowing: 2.7%
Koroulakis, 2021^[29]^	Rectal cancer	NA	28	16–63 Gy	1- year was 81.8%	1- year was 45.0%	NA	Acute, grade 3 gastrointestinal and skin-related: 10.7% Late, grade 3 and above, e.g., presacral abscess/bleeding, rectovaginal fistula: 14.2%

## Application of Proton Therapy in the Treatment of Head and Neck Cancers

Currently, in the treatment of head and neck cancers, surgery is primarily utilized, often in combination with radiotherapy. In cases where surgery is not feasible or in recurrent cases, radiotherapy alone is employed. Due to the proximity of critical organs at risk (OARs) (such as the brain, brainstem, eyes, and cranial nerves), the radiation dose must be strictly limited [30]. Proton therapy, owing to its physical properties, has the ability to reduce the total delivered dose, thereby decreasing patient side effects while maintaining effectiveness [31].

### Tonsillar Cancer

A study conducted in Sweden among patients with tonsillar cancer demonstrated that delivering a higher dose through proton therapy compared to conventional radiotherapy yields better outcomes in controlling the local focus of the tumor and improves patient survival [2]. Additionally, proton therapy delivers a lower dose to healthy structures adjacent to the tumor site, resulting in improved quality of life and a reduced risk of complications. Furthermore, it has been shown that the physical properties of proton beams make radiation particularly beneficial for orbital tumors, periorbital regions, and the nasal area [2].

### Mucosal Melanoma

A large study conducted by the Massachusetts General Hospital team and published in 2008 involved 102 patients with head and neck tumors of various histological types treated surgically (where feasible) between 1991 and 2002, followed by adjuvant therapy combining photon and proton therapy [30]. An important finding of this study was the high rate of local control and survival in patients with inoperable mucosal melanoma treated with proton therapy, which resulted in a reduced toxicity rate compared to data on photon therapy [30].

Another multicenter phase II study on proton therapy for nasal cavity and paranasal sinus mucosal melanoma was conducted from June 2008 to October 2012 at 2 hospitals, the National Cancer Center Hospital East and Tsukuba University Hospital, where 32 patients with histologically confirmed mucosal melanoma in stage N0M0 were enrolled [32]. The study showed a local tumor control rate of 75.8%, with 3-year survival rates of 46.1%. Compared to the standard approach in treating mucosal melanoma of the head and neck, which involves surgical treatment with postoperative radiotherapy and a 5-year survival rate ranging from 20 to 45%, proton therapy was found to provide sufficient local control in locally advanced cases. The main cause of death among the study participants was tumor-related mortality due to distant metastases, suggesting that local improvement with proton therapy alone is insufficient to inhibit distant metastases [32].

### HPV-Positive Head and Neck Cancer

Two distinct clinical and biological types of squamous cell head and neck cancer have been identified: one associated with tobacco smoking and alcohol consumption, and the other associated with Human papillomavirus (HPV) infection, the prevalence of which is increasing, especially among young non-smokers and non-drinkers [33]. This patient group has fewer comorbidities, more often has a lower T stage but a more advanced N stage, and has a better prognosis compared to patients with HPV-negative disease [31].

Avoiding long-term adverse effects of radiotherapy is particularly important among young patients with HPV-positive oral and throat cancer, who have better disease control outcomes, resulting in longer survival even with the presence of radiation-related side effects and complications requiring prolonged treatment and compromising their quality of life [31].

Therefore, a laboratory study was conducted by The University of Texas using squamous cell head and neck cancer cell lines [33]. The study found that HPV-positive cell lines were more sensitive to proton therapy than HPV-negative lines, and DNA double-strand damage in HPV-positive cells was more durable. Hence, positive HPV testing in head and neck tumors may be a marker of sensitivity to proton therapy [33].

A unit described a dosimetric comparison of 25 patients with oral and throat cancer treated with IMPT. It was found that the mean doses delivered to the oral cavity, hard palate, larynx, jaw, esophagus, and central nervous system centers responsible for nausea and vomiting in patients were significantly lower with intensity-modulated proton therapy (IMPT) than with intensity modulated radiation therapy (IMRT) comparative plans generated for the same patients [31].

Another study was conducted from 2010 to 2024 at the same medical facility [31]. The study included 50 IMPT patients and 100 IMRT patients. The median observation time was 32 months. The study found no significant therapeutic difference between the two methods: intensity modulated radiation therapy (IMRT) and intensity-modulated proton therapy (IMPT). However, the use of IMPT resulted in a reduction in the number of gastric tubes during the acute phase of treatment, as well as at 3 and 12 months after IMPT completion, resulting in a reduction in severe weight loss and improvement in patient quality of life [31].

The Hyogo Ion Beam Medical Center was the world's first facility established in Japan in 2001, where both proton therapy and carbon-ion radiotherapy were utilized [34]. A study was conducted at this center involving 208 patients with unresectable or radiation-resistant head and neck tumors, of whom 167 patients underwent proton therapy and 41 patients received carbon-ion therapy. Approximately 78% of the study participants had stage T3/T4 tumors. The overall survival rate was 37.3%, while the 5-year local control rate was 65.9%. The disparity between these rates stems from the fact that the main cause of death among the study participants was distant metastases associated with tumor stage. However, in the case of malignant melanoma, squamous cell carcinoma, and adenocarcinoma, not only metastases but also local growth led to a decrease in survival rates. Most patients in the control group had locally advanced tumors resistant to radiotherapy. The most significant finding of the study was that patients with radiotherapy-resistant adenocystic carcinoma achieved very good local control (92% after 3 years of the study). The study results were highly encouraging for the use of proton therapy in patients with head and neck tumors due to its excellent local control and minimal functional and cosmetic damage [34].

## Proton Therapy—Toxicity, Quality of Life, and Treatment Cost-effectiveness

Proton therapy allows for the delivery of radiation doses to critical organs such as the eyes, optic chiasm, and brainstem to be reduced, resulting in a decrease in the occurrence of late adverse effects on these organs [2]. The occurrence of side effects caused by proton therapy depends on the relative and absolute volume of organs at risk (OAR) receiving specific radiation doses [14].

Studies conducted in Sweden indicate low doses delivered to salivary glands, which is associated with a reduced risk of xerostomia, leading to a decrease in the number of dental procedures and an increase in the Quality-adjusted life year (QALY) index [2].

In a study conducted among 50 patients with oral and throat cancer observed over 3 years, the following results regarding adverse effects after proton therapy were obtained [35]. Acute adverse effects were dominated by skin inflammation, mucositis, and dysphagia. During therapy, 32% of patients visited the Emergency Department, mostly due to dehydration and pain caused by mucositis of the oral cavity accompanied by odynophagia. Special attention was also paid to the reduction in the number of gastric tubes inserted (27%) compared to IMRT (47%). All gastric tubes were removed during follow-up visits, on average 2 months after placement, with no observed long-term or advanced swallowing problems or esophageal strictures in these patients. Prophylaxis ("eat and exercise") implemented during proton therapy, which involved maximizing the swallowing process, also reduced the number of functional adverse effects [35]. Another study also showed a reduction in the number of gastric tubes inserted during and after IMPT, resulting in a reduction in the number of patients with significant weight loss and an increase in the QALY index [31].

In a retrospective study conducted at The University of Texas, 584 patients with throat and oral cancer were considered, of whom 534 were treated with IMRT and 50 with IMPT, with cases evaluated for the occurrence of osteoradionecrosis as a complication of therapy [36]. The study showed that 41 out of 534 patients (7.7%) treated with IMRT developed osteoradionecrosis, while in the IMPT group, osteoradionecrosis of grade 1 occurred in only 1 patient. This study also showed that the radiation dose delivered to the jaw was smaller regardless of the dose-volume ratio, clearly indicating the superiority of IMPT therapy in the treatment of oral and throat cancer [36].

Considering the high costs of proton therapy, it is important to determine whether proton therapy will be a better therapeutic solution for a given patient. In Denmark, an online platform called PRODECIS (PROton DECIsion Support) was created to assist doctors in choosing between proton therapy and conventional radiotherapy for individual patients. This platform compares treatment methods based on their toxicity, dose used, and cost-effectiveness levels. In this study, it was tested among patients with head and neck cancer (HNC) [37].

In the selected patient group, adverse effects of proton therapy were analyzed. The average risk of dysphagia 6 months after treatment decreased from 37 to 28% and from 23 to 18% after 12 months. The risk of xerostomia after treatment decreased in all 23 cases: from 48 to 25% after 6 months and from 46 to 23% after 12 months [37].

In the assessment of the cost-effectiveness of proton therapy among patients in this study, the QALY index was used, calculated by estimating the quality and quantity of life extended by medical intervention. 1 QALY point obtained by the patient corresponded to €80,000 spent during therapy. Through such an assessment, it was found that proton therapy was cost-effective in 8 out of 23 cases among the patient group [37].

The study shows that considering the guidelines adopted in Denmark for reducing the profile of adverse effects of proton therapy by 15%, including dysphagia and xerostomia, all patients would benefit from proton therapy after 6 months, and 91% after 12 months, while 35% could be considered cost-effective at the established threshold of €80,000 per QALY point [37].

## Conclusion

Proton therapy represents a promising alternative to conventional radiotherapy due to the reduced number of complications in healthy tissues by delivering a lower radiation dose outside the tumor area. This is particularly important in the young patient group, such as HPV-positive individuals with head and neck tumors, who have a better prognosis and longer survival, thus their quality of life after treatment is crucial. The article cites numerous studies confirming the effectiveness and wide application of proton therapy. However, more research is needed to assess the long-term toxicity of proton therapy compared to conventional radiotherapy.

## Data Availability

No datasets were generated or analysed during the current study.
